# Cannabidiol alleviates carbon tetrachloride-induced liver fibrosis in mice by regulating NF-κB and PPAR-α pathways

**DOI:** 10.3389/ebm.2024.10141

**Published:** 2024-04-22

**Authors:** Run Ma, Na Xie, Yuanhui Shu, Yafeng Wu, Ping He, Yining Xiang, Yan Zhou, Yuping Wang

**Affiliations:** ^1^ Center for Clinical Laboratories, The Affiliated Hospital of Guizhou Medical University, Guiyang, Guizhou, China; ^2^ School of Clinical Laboratory Science, Guizhou Medical University, Guiyang, Guizhou, China; ^3^ Sichuan Provincial Center for Disease Control and Prevention, Chengdu, Sichuan, China; ^4^ Clinical Laboratory, The Fourth People’s Hospital of Ya’an City, Ya’an, Sichuan, China; ^5^ Pathology, The Affiliated Hospital of Guizhou Medical University, Guiyang, Guizhou, China

**Keywords:** cannabidiol, liver fibrosis, carbon tetrachloride, NF-κB, PPAR-α

## Abstract

Liver fibrosis has become a serious public health problem that can develop into liver cirrhosis and hepatocellular carcinoma and even lead to death. Cannabidiol (CBD), which is an abundant nonpsychoactive component in the cannabis plant, exerts cytoprotective effects in many diseases and under pathological conditions. In our previous studies, CBD significantly attenuated liver injury induced by chronic and binge alcohol in a mouse model and oxidative bursts in human neutrophils. However, the effects of CBD on liver fibrosis and the underlying mechanisms still need to be further explored. A mouse liver fibrosis model was induced by carbon tetrachloride (CCl_4_) for 10 weeks and used to explore the protective properties of CBD and related molecular mechanisms. After the injection protocol, serum samples and livers were used for molecular biology, biochemical and pathological analyses. The results showed that CBD could effectively improve liver function and reduce liver damage and liver fibrosis progression in mice; the expression levels of transaminase and fibrotic markers were reduced, and histopathological characteristics were improved. Moreover, CBD inhibited the levels of inflammatory cytokines and reduced the protein expression levels of p-NF-κB, NF-κB, p-IκBα, p-p38 MAPK, and COX-2 but increased the expression level of PPAR-α. We found that CBD-mediated protection involves inhibiting NF-κB and activating PPAR-α. In conclusion, these results suggest that the hepatoprotective effects of CBD may be due to suppressing the inflammatory response in CCl_4_-induced mice and that the NF-κB and PPAR-α signaling pathways might be involved in this process.

## Impact statement

In this study, CBD had a hepatoprotective effect on CCl_4_-induced liver fibrosis in mice by increasing antioxidant effects, and its mechanism of action may be related to the regulation of the NF-κB and PPAR-α signaling pathways. Therefore, CBD and related compounds could represent novel pharmacological agents to treat fibrosis.

## Introduction

Liver fibrosis is mainly caused by the progression of various chronic liver diseases. Worldwide, chronic liver diseases mainly include alcoholic hepatitis, nonalcoholic steatohepatitis (NASH), nonalcoholic fatty liver disease (NAFLD), viral hepatitis [hepatitis B (HBV) and hepatitis C (HCV)] and cholestatic liver disease [1–3]. In liver fibrosis, the most important pathological change in the liver is the formation and deposition of extracellular matrix (ECM) [4, 5]. The amount of type I collagen (COL-I) deposition positively correlates with the severity of fibrosis [6, 7]. The activation of hepatic stellate cells (HSCs), which express alpha-smooth muscle actin (α-SMA) and secrete and synthesize ECM and various autocrine or paracrine cytokines, such as tumor necrosis factor-α (TNF-α), interleukin 1β (IL-1β) and interleukin 6 (IL-6), is a crucial link in the occurrence of liver fibrosis. Many intracellular signaling pathways, including the transforming growth factor-β1 (TGF-β1) [8–10] and nuclear factor kappa B (NF-κB) [11, 12] pathways, are involved in HSC activation.

Hepatic injury involves stress signaling [e.g., mitogen-activated protein kinase p38 (p38MAPK) and c-Jun N-terminal kinase (JNK)] [13] and proinflammatory pathways [e.g., NF-κB [14, 15] and cyclooxygenase-2 (COX2)] [16] in various cell types, which in turn modulate important inflammatory and cell death processes. NF-κB is a key regulator that stimulates the expression of inflammatory factors, chemokines and adhesion molecules and plays an important role in cell growth, differentiation and apoptosis. COX-2 is an important enzyme in the synthesis of prostaglandins from arachidonic acid and plays a key role in the inflammatory response [17]. The interaction of peroxidase proliferator-activated receptor-α (PPAR-α) with other signaling pathways can also regulate cellular redox status. For example, PPAR-α activation can inhibit the transcription of NF-κB and oxidative stress and reduce the release of inflammatory cytokines [18].

Cannabidiol (CBD) is the main nonpsychoactive component of the cannabis plant and has many beneficial pharmacological effects, such as anti-inflammatory and antioxidant effects [19–21]. Studies have shown that CBD can be used for alcohol use disorder (AUD) and alcohol-related damage to the brain and liver [22]; CBD can be used to treat conditions such as colitis, arthritis and type 1 diabetes, alcohol-induced lipodystrophy, or hypoxia-ischemia-induced brain damage [23, 24]; and CBD greatly alleviates liver inflammation, oxidative/nitrative stress, and cell death and inhibits bacterial endotoxin-induced NF-κB activation and TNF-α production in Kupffer cells [25]. Currently, there are limited treatment options for liver fibrosis, and it is of great clinical importance to identify drugs that can prevent fibrosis progression or even reverse it. This study examined the protective effect of CBD in CCl_4_-induced mice with liver fibrosis, which involved the NF-κB and PPAR-α signaling pathways, and revealed its potential mechanism of action.

## Materials and methods

### Experimental reagents and equipments

The following reagents were used: CBD (Sigma, United States); colchicine (Beyotime, China); CCl_4_ (Aladdin, China); peanut oil (Yuanye, China); ELISA kits (Novus, United States); hematoxylin and eosin (HE) and Masson assay kits (Beyotime, China); aspartate aminotransferase (AST) and hyaluronic acid (HA) kits (Nanjing Jiancheng Bioengineering Institute, China); RIPA lysis buffer and a BCA kit (Solaibao, China); COX-2, p-IκBα, and IκBα antibodies (Wanleibio, China); p-NF-κB, NF-κB, p-p38 MAPK, and p-38 MAPK antibodies (CST, United States); TGF-β1, α-SMA, COL-I, PPAR-α and GAPDH antibodies (Abcam, United States); horseradish peroxidase-labeled secondary antibodies (Bioprimacy, China); chemiluminescence (ECL) color developing solution (Merck, United States); an RNeasy mini kit (Axygen, United States); a PrimeScript RT reagent kit with gDNA Eraser and SYBR Green Master Mix (Takara, Japan); an automatic chemical analyzer (Hitachi, Japan); and light microscopy (Nikon, Japan).

### Experimental animals and treatments

Forty mice (C57BL/6J) were purchased from Guizhou Medical University. All animal experiments were approved by the Guizhou Medical University Animal Care Welfare Committee. Male (6–8 weeks old) mice weighing approximately 20 g were adapted to the animal environment, and food and water were available randomly. One week later, the mice were randomly divided into five groups with the following injection for 10 weeks: (Ⅰ) In the control group, normal mice were treated with a peanut oil solution twice weekly (*n* = 8). (Ⅱ) In the CCl_4_ group, mice were intraperitoneally administered a 30% CCl_4_ peanut solution (5 mL/kg) twice weekly (*n* = 8). (Ⅲ) In the 4 mg/kg CBD group, mice were administered 4 mg/kg CBD intraperitoneally and the same CCl_4_ as the CCl_4_ group (*n* = 8). (Ⅳ) In the 8 mg/kg CBD group, mice were administered 8 mg/kg CBD intraperitoneally and the same CCl_4_ as the CCl_4_ group (*n* = 8). (V) In the colchicine group, mice were administered 0.2 mg/kg colchicine intraperitoneally and the same CCl_4_ peanut solution as the CCl_4_ group (*n* = 8). Two hours before each intraperitoneal injection of CCl_4_, the mice in the CBD and colchicine groups were intraperitoneally injected with the appropriate drug at the indicated dose (4, 8 mg/kg CBD, 0.2 mg/kg colchicine), and mice in the control group and model group were injected with normal saline. All mice were euthanized by anesthesia 24 h after the last administration, and blood and liver tissues were collected for further examination. The left lobe of the liver was quickly fixed in 4% paraformaldehyde, and the remaining liver samples were collected, frozen in liquid nitrogen and stored at −80°C.

### Serum biochemical analysis

Serum was obtained by centrifuging (3,000 × g, 4°C, for 10 min) whole blood. AST and HA levels were determined using a clinical chemical analyzer.

### ELISA analysis of inflammatory cytokines

Liver tissues were promptly removed and washed in precooled normal saline. Liver tissues (0.1 g) were completely homogenized with cold saline (0.9 mL) by sonication on ice to obtain 10% tissue homogenate, and the supernatant was obtained by centrifugation. The Coomassie brilliant blue method was used to measure the total protein content, and the level of IL-1β was measured using the corresponding assay kit. Serum levels of IL-6 and TNF-α were measured by Valukine ELISA kits according to the manufacturer’s instructions.

### Examination of pathological changes

Fresh liver samples were fixed in 4% paraformaldehyde for 24 h and paraffin-embedded tissue sections (4 µm). The extent of inflammation and cell necrosis in the liver was observed using HE staining. Liver fibrosis was effectively and widely evaluated at the histological level by staining collagen fibers with Masson’s trichrome staining. The histological degree of liver fibrosis was observed using light microscopy by a pathologist who was blinded to this test.

### Immunohistochemical staining

The sections were dewaxed, hydrated, boiled in citrate buffer for 5 minutes, cooled to room temperature, and washed three times in PBS buffer. Next, sections were incubated in 3% H2O2 for 10 min to block endogenous peroxidase activity and were blocked with 10% bovine serum in a water bath at 37°C for 30 min to block nonspecific binding. Immunohistochemistry was performed by incubating the sections overnight at 4°C with primary antibodies (1/1000) (α-SMA and COL- I) and rinsing the sections three times with PBS. Biotinylated secondary antibodies were added according to the kit instructions. The sections were incubated with the DAB reagent for 3 min to develop the color, and the sections were counterstained with Mayer’s hematoxylin. Finally, the sections were dehydrated in ethanol, cleared in xylene and sealed with neutral gum. Specific primary antibodies were substituted with PBS or nonimmune isotype-matched sera as the negative control. Images were captured by light microscopy, five fields were randomly selected, and the percentage of the area was assessed by ImageJ.

### Western blotting

The liver tissue was washed with precooled saline and then dried with filter paper. Protein samples were obtained from liver tissues in RIPA lysis solution with sonication on ice, and then the protein concentration was determined by the BCA method. A total of 30 µg of protein was used for western blot analysis. Protein samples were separated by 10% SDS-PAGE and then transferred to PVDF membranes. The PVDF membranes were incubated with the appropriate primary antibodies (TGF-β1 1/1000, α-SMA 1/10,000, COL-I 1/1000, p-NF-κB 1/500, NF-κB 1/1000, p-p38 MAPK 1/1000, p38 MAPK 1/1000, p-IκBα 1/500, IκBα1/500, COX-2 1/1000, PPAR-α 1/500, and GAPDH 1/10,000) at 4°C overnight and then with the corresponding horseradish peroxidase-conjugated secondary antibodies. Next, the chromogenic solution was added dropwise, and the corresponding protein bands were detected after exposure. ImageJ software was used to measure the gray values of the bands.

### Quantitative real-time polymerase chain reaction (PCR)

Liver tissues (0.1 g) were homogenized, and mRNA was isolated using the RNeasy mini kit following the manufacturer’s instructions. Reverse transcription was performed using the PrimeScript RT reagent kit with gDNA Eraser and SYBR Green Master Mix. Real-time PCR was performed for each sample using a Roche Real-Time PCR System. Transcription specificity was confirmed by melting curve profiles generated at the end of the PCR program. The data are expressed as the expression of the target genes normalized to the expression of GAPDH and were quantified using the comparative cycle threshold Ct method (2^−ΔΔCT^) (Table 1).

**TABLE 1:** Primer sequences for quantitative real-time polymerase chain reaction.

Gene	Forward primer (5′-3′)	Reverse primer (5′-3′)
GAPDH	AAG​AAG​GTG​GTG​AAG​CAG​GCA​TC	CGG​CAT​CGA​AGG​TGG​AAG​AGT​G
COL-Ⅰ	ACG​CCA​TCA​AGG​TCT​ACT​GC	ACT​CGA​ACG​GGA​ATC​CAT​CG
α-SMA	GCC​ATC​TTT​CAT​TGG​GAT​GGA	CCC​CTG​ACA​GGA​CGT​TGT​TA
TGF-β	GCT​GAA​CCA​AGG​AGA​CGG​AA	ATG​TCA​TGG​ATG​GTG​CCC​AG
IL-1β	CTTCAGGCAGGCAGTATC	CAG​CAG​GTT​ATC​ATC​ATC​ATC
IL-6	CCC​CAA​TTT​CCA​ATG​CTC​TCC	CGC​ACT​AGG​TTT​GCC​GAG​TA
TNF-α	TCA​GTT​CCA​TGG​CCC​AGA​C	GTT​GTC​TTT​GAG​ATC​CAT​GCC​ATT

### Statistical analysis

Values are expressed as the mean ± standard deviation. The statistical significance of differences was determined using Student’s *t*-test (for two groups) or one-way ANOVA (for more than two groups) followed by the LSD multiple comparisons test. All analyses were performed with PASW statistics 26 (SPSS). *p* < 0.05 was considered significant.

## Results

### CBD treatment ameliorated liver damage

**FIGURE 1:**
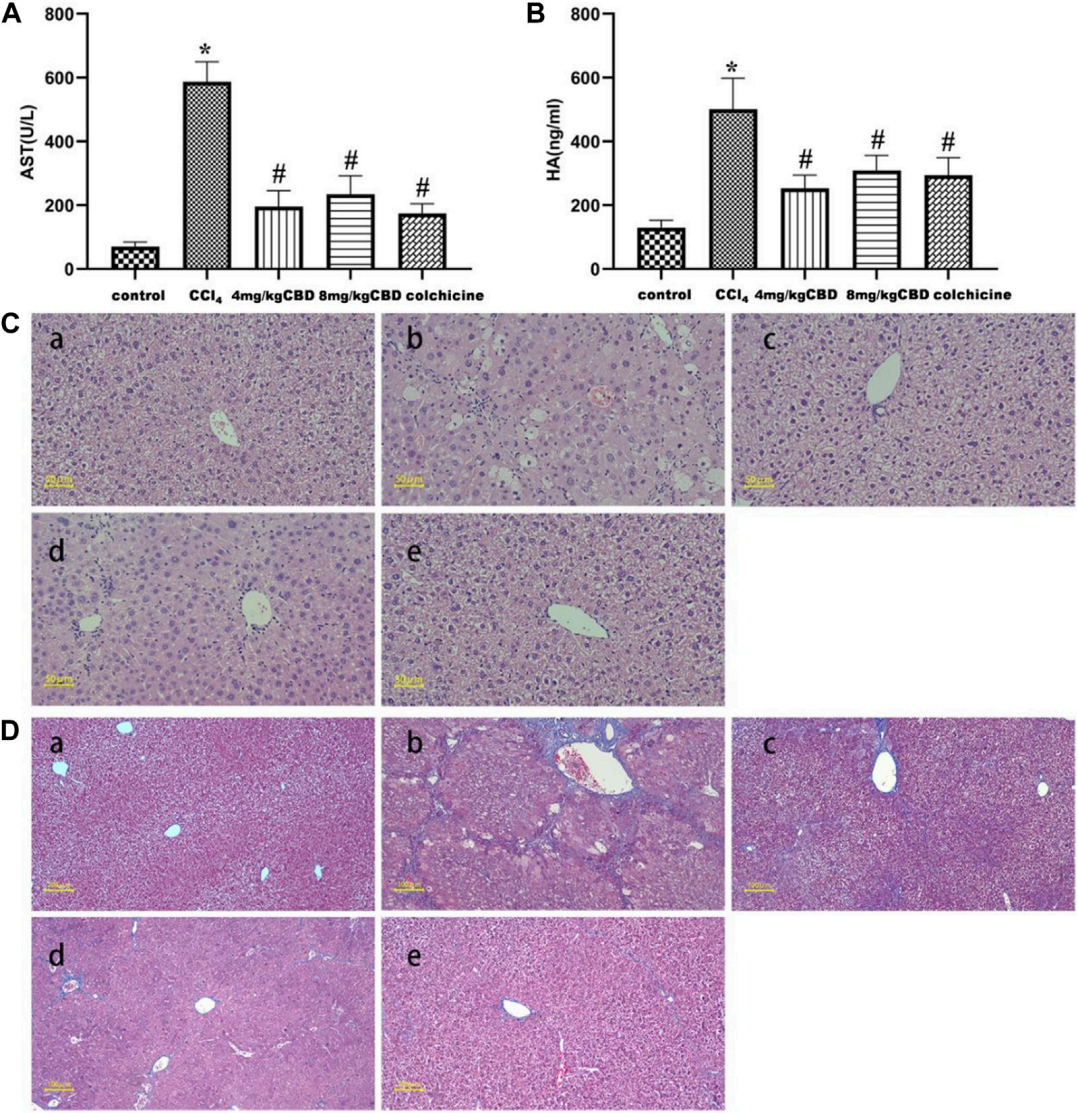
Effects of CBD treatment on liver function in mice with CCl_4_-induced liver fibrosis. **(A,B)** Serum AST and HA levels in the indicated groups. **(C)** Representative HE staining of liver sections. **(D)** Fibrosis deposition was observed by Masson trichrome staining. The values represent the means ± SEM (*n* = 8). ^*^ *p < 0.05* vs. the control group, ^*#*^ *p < 0.05* vs. the CCl_4_ group, as determined by one-way ANOVA, followed by the LSD multiple comparisons test. (a) Control group; (b) CCl_4_ group; (c) 4 mg/kg CBD group; (d) 8 mg/kg CBD group; (e) 0.2 mg/kg colchicine group.

The levels of AST and HA are considered important markers for evaluating liver function. We found that CCl_4_ significantly increased the levels of AST and HA compared to those in the control group; the administration of CCl_4_ to mice treated with CBD and colchicine markedly reduced the levels of AST and HA. However, no significantly different expression levels of AST and HA were found after 4 mg/kg and 8 mg/kg CBD injection (Figures 1A, B). Liver injury was assessed by HE staining. In the liver tissue of the control group, liver cells were neatly arranged and showed no necrosis, indicating normal liver tissue structure. In the CCl_4_ group, hepatocyte disorder, necrosis, swelling, and a large amount of inflammatory cell infiltration indicated serious liver damage. Compared with the CCl_4_ group, the CBD and colchicine groups had significantly reduced liver damage, with only mild necrosis and a relatively stable cell structure (Figure 1C). Masson staining showed that the liver tissue of the control group exhibited a normal lobular structure, the radial hepatic cords were neatly arranged, and there was no collagen accumulation around the blood vessels; the structure of the liver lobules in the CCl_4_ group was severely damaged, a large amount of collagen was deposited, and pseudo lobules had formed, indicating that the model of liver fibrosis was successfully established; and in the CBD and colchicine groups, the liver was not significantly damaged, the accumulation of collagen was reduced, and the degree of fibrosis was significantly improved compared with that in the CCl_4_ group. The histological changes observed in both fibrosis models were significantly attenuated by CBD (Figure 1D). The extent of the improvement did not increase with the CBD dose. Combined with the serological results, these data showed that CBD had hepatoprotective effects on CCl_4_-induced mice.

### CBD treatment attenuated CCl_4_-induced liver fibrosis

**FIGURE 2:**
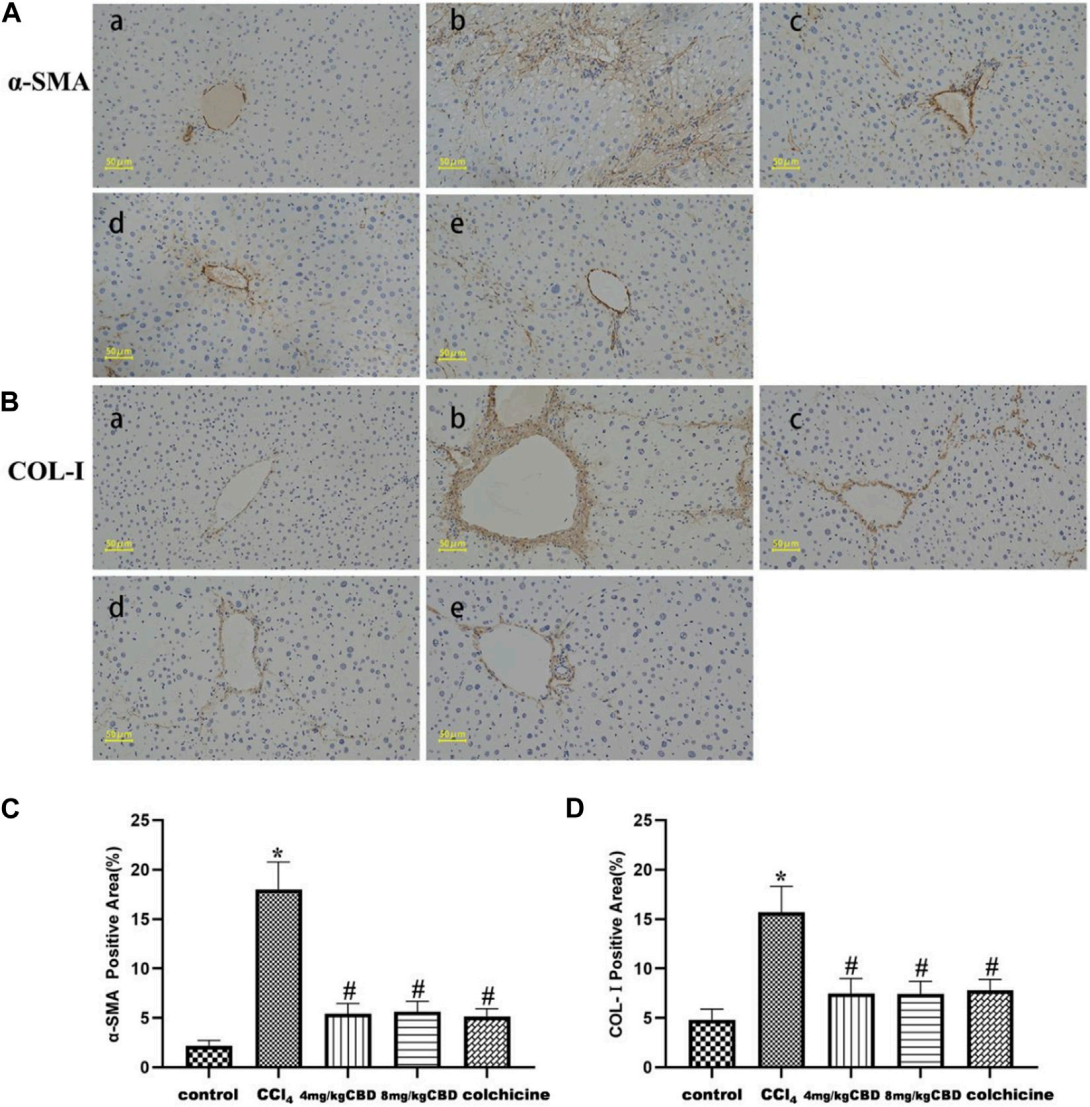
Effects of CBD on α-SMA and COL-Ⅰ in mice with CCl_4_-induced liver fibrosis. **(A,B)** Representative immunohistochemical staining of α-SMA and COL-Ⅰ. **(C,D)** Quantification of positive staining areas was performed by ImageJ software. The values represent the means ± SEM (*n* = 8). ^*^ *p < 0.05* vs. the control group, ^*#*^ *p < 0.05* vs. the CCl_4_ group, as determined by one-way ANOVA, followed by the LSD multiple comparisons test. (a) Control group; (b) CCl_4_ group; (c) 4 mg/kg CBD group; (d) 8 mg/kg CBD group; (e) 0.2 mg/kg colchicine group.

In the liver tissue of the control group, α-SMA and COL-Ⅰ were weakly expressed in the portal area and central vasculature wall; in the CCl_4_ group, α-SMA, and COL-Ⅰ were widely expressed, mainly distributed in the portal ducts of fibrous tissue proliferation and the interarea interval, and diffuse expression was also observed between the central vein and hepatocytes; and the sites of α-SMA and COL-Ⅰ expression in the CBD group and colchicine groups were the same as those in the CCl_4_ group, but the areas were markedly reduced (Figures 2A, B). Positive area expression analysis also showed similar decreases in α-SMA and COL-Ⅰ after CBD administration. However, no significant differences in the expression of α-SMA and COL-Ⅰ were found between 4 mg/kg and 8 mg/kg CBD (Figures 2C, D).

### CBD treatment alleviated the production of inflammatory mediators

**FIGURE 3:**
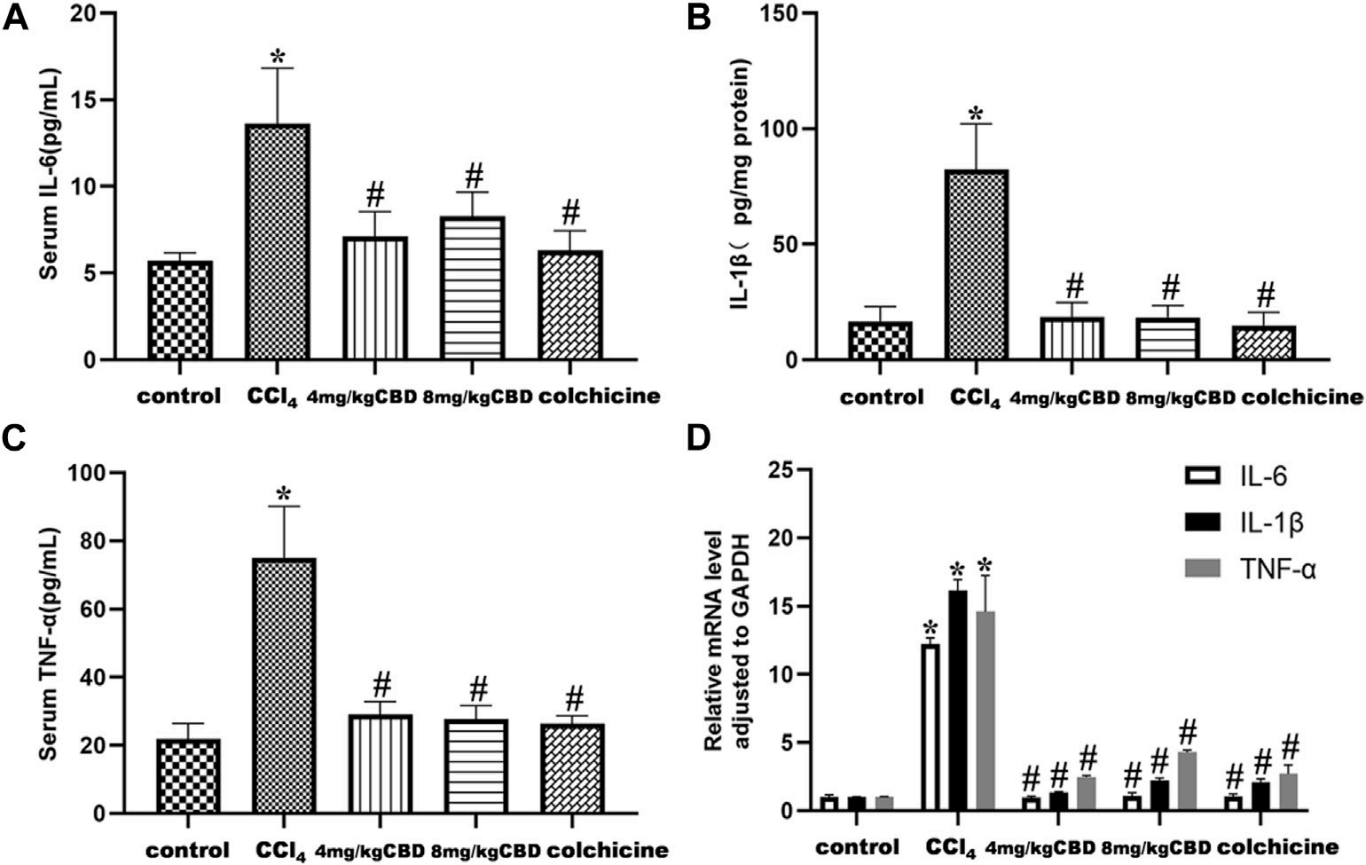
Effects of CBD on inflammatory cytokines in mice with CCl_4_-induced liver fibrosis. **(A–C)** Serum levels of IL-6 and TNF-α and liver levels of IL-1β. **(D)** The mRNA levels of IL-6, IL-1β and TNF-α were measured by q-PCR. The values represent the means ± SEM (*n* = 8). ^*^ *p < 0.05* vs. the control group, ^*#*^ *p < 0.05* vs. the CCl_4_ group, as determined by one-way ANOVA, followed by the LSD multiple comparisons test.

Inflammatory cytokines play a pivotal role in liver fibrosis. The levels of the inflammatory markers IL-6, IL-1β, and TNF-α in the different treatment groups were measured by ELISA. CCl_4_ treatment markedly elevated the levels of serum IL-6 and TNF-α and liver IL-1β compared with those in the control group. Treatment with CBD significantly reversed the increases in the expression of IL-6, IL-1β, and TNF-α, and the same results were obtained after colchicine treatment (Figures 3A–C). Semiquantitative q-PCR analysis also showed similar decreases in IL-6, IL-1β, and TNF-α mRNA expression after CBD administration. The levels of the inflammatory markers IL-6, IL-1β, and TNF-α were not significantly different between 4 mg/kg and 8 mg/kg CBD (Figure 3D).

### CBD treatment attenuated the expression levels of TGF-β1, α-SMA, and COL-I

**FIGURE 4:**
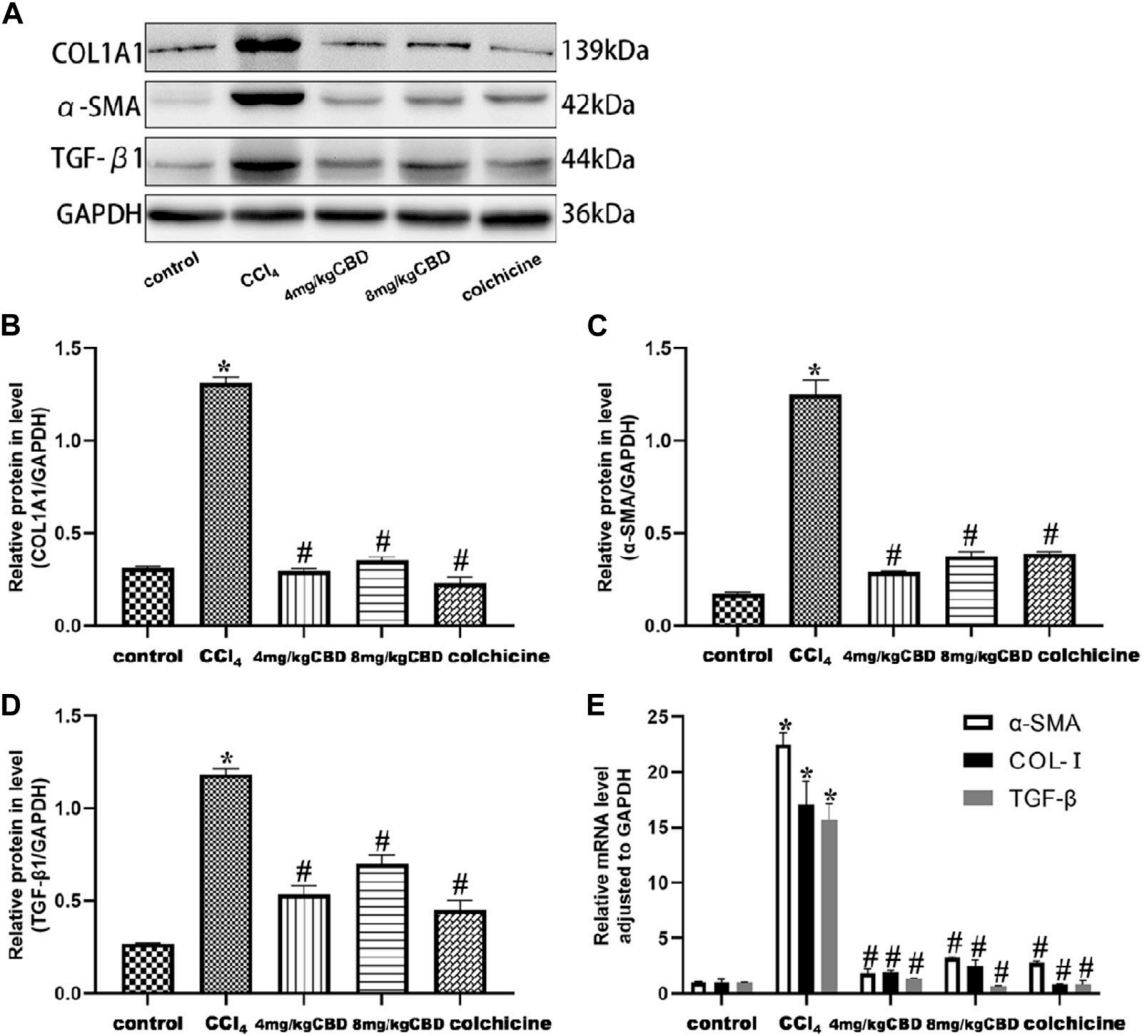
Effects of CBD on TGF-β1, α-SMA, and COL- I in mice with CCl_4_-induced liver fibrosis. **(A)** The effects of CBD on the protein expression of TGF-β1, α-SMA, and COL- I were measured by western blotting. **(B–D)** ImageJ software was used to measure the grey values of the bands. **(E)** The effect of CBD on the mRNA expression of TGF-β1, α-SMA, and COL- I was determined by q-PCR. The values represent the means ± SEM (*n* = 8). ^*^ *p < 0.05* vs. the control group, ^*#*^ *p < 0.05* vs. the CCl_4_ group, as determined by one-way ANOVA, followed by the LSD multiple comparisons test.

In response to CCl_4_, the expression levels of TGF-β1, α-SMA, and COL-I in the liver in the CCl_4_ group increased significantly, and these expression levels were effectively reduced by CBD and colchicine (Figures 4A–D). The semiquantitative q-PCR results showed increased expression of TGF-β1, α-SMA and Col-Ⅰ in liver tissues in the CCl_4_ group compared with the control group; compared with that in the CCl_4_ group, the expression of TGF-β1, α-SMA and Col-Ⅰ markedly decreased after treatment with CBD and colchicine (Figure 4E). The western blot and Semiquantitative q-PCR results showed that the expression levels of TGF-β1, α-SMA, and COL-I were similar between 4 mg/kg and 8 mg/kg CBD. Importantly, the results showed that CBD treatment attenuated CCl_4_-induced liver fibrosis.

### CBD treatment protected the liver by inhibiting the NF-κB pathway and activating the PPAR-α pathway

**FIGURE 5:**
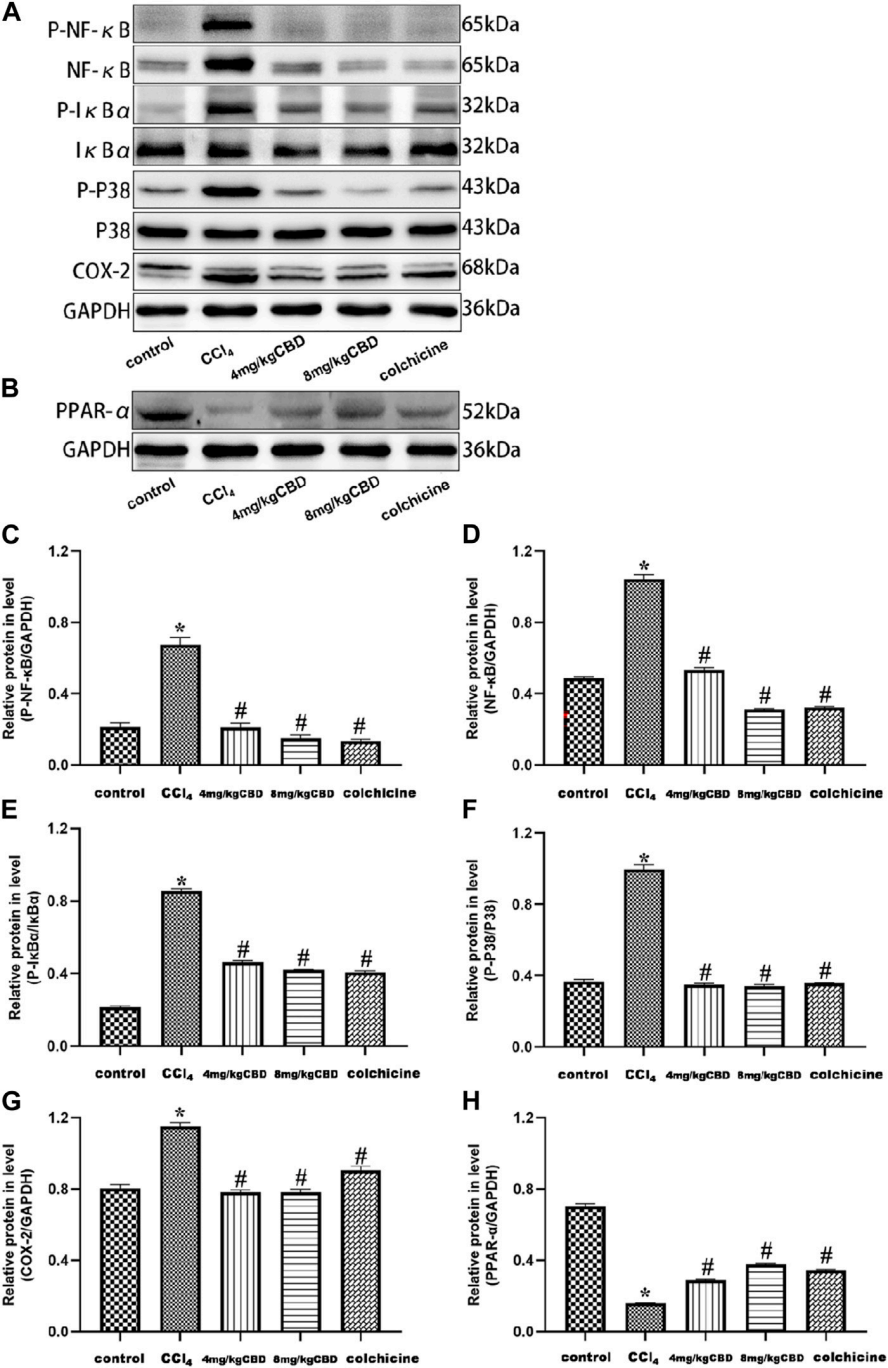
Effects of CBD on NF-κB- and PPAR-α-related proteins in mice with CCl_4_-induced liver fibrosis. **(A,B)** The effects of CBD on the protein expression levels of NF-κB, p-NF-κB, p-IκBα, IκBα, p-p38 MAPK, p38 MAPK, COX-2, and PPAR-α were measured by western blotting. **(C–H)** ImageJ software was used to measure the grey values of the bands. The values represent the means ± SEM (*n* = 8). ^*^ *p < 0.05* vs. the control group, ^*#*^ *p < 0.05* vs. the CCl_4_ group, as determined by one-way ANOVA, followed by the LSD multiple comparisons test.

To explore the potential mechanism of CBD mediated protection, we measured the expression of NF-κB and PPAR-α related proteins in liver tissues by western blotting. Compared with those in the control group, the expression levels of p-NF-κB, NF-κB, and COX-2 and the p-IκBα/IκBα and p-p38/p38 ratios in the CCl_4_ group were significantly increased. Interestingly, CBD and colchicine treatment reversed these alterations in liver fibrosis, suggesting that the effect of CBD on CCl_4_-induced inflammation was associated with inhibiting the activation of the NF-κB pathway. In addition, PPAR-α was highly expressed in the control group, and the expression of PPAR-α in the liver was significantly decreased in the CCl_4_ group compared with the control group. CBD and colchicine treatment significantly increased the expression of PPAR-α in the liver, indicating that CBD may modulate PPAR-α signaling in mice with CCl_4_-induced liver fibrosis (Figures 5A–H).

## Discussion

Liver fibrosis is the pathological result of abnormal ECM accumulation in the liver and is closely related to hepatic morbidity and mortality [26]. Some studies have shown that the development of liver fibrosis can be prevented by experimental clinical treatments, but many patients do not have good reactions [27]. Therefore, there is an urgent need for new therapeutic approaches to reverse fibrosis. CBD is one of the main components extracted from cannabis, and its level is second only to that of tetrahydrocannabinol (THC) [19]. In recent years, the medicinal value of CBD has become increasingly apparent, and long-term use has shown good tolerance and no side effects in humans [28, 29]. CBD can regulate the immune system in different tissues and reduce oxidative/nitrative stress [30], cell death and inflammatory responses, such as IL-6, COX2, and NF-κB, neutrophil infiltration, and stress signaling [31–33]. Researchers have found that CBD has some important effects on the central nervous system, including antipsychotic, antianxiety, antiepileptic, and analgesic effects [34]. In addition, CBD also has complex immune regulation, anti-inflammatory and antioxidant effects [35]. Studies have reported that CBD can induce apoptosis of thymus cells and spleen cells, inhibit the proliferation of T cells and macrophages, and have certain therapeutic effects on autoimmune diseases [36, 37]. CBD can inhibit T-cell-mediated chronic autoimmune myocarditis and myocardial reconstruction/fibrosis and improve myocardial dysfunction [38]. CBD induces functional Treg cells to induce immunosuppression under low-level T-cell stimulation [39]. CBD is used to treat brain damage caused by colitis, diabetic complications, drug-induced nephrotoxicity, alcohol fat deposition or hypoxic-ischemia [40–42]. Treatment of mice with cannabidiol markedly attenuated the cisplatin-induced oxidative/nitrosative stress, inflammation, and cell death in the kidney, and it improved renal function [43]. In the prevention and treatment of skin and liver fibrosis, CBD inhibited collagen gene transcription and synthesis and prevented TGF-β and IL-4 induced fibroblast migration [44]. CBD treatment decreases the inflammatory and remodelling processes in a murine model of ovalbumin induced allergic asthma [45]. Previous studies have examined whether CBD has a protective effect on alcoholic fatty liver disease [46], but its effect on fibrosis and the detailed mechanisms in the context of inflammation remain unclear. We analyzed the effects of CBD on CCl_4_-induced liver inflammation and collagen deposition using a well-established model that is very similar to human liver fibrosis [47, 48]. Colchicine, which is an alkaloid agent that is generally used to treat acute gout in the clinic, was used as the positive control for its effects on improving liver fibrosis and ameliorating liver function [49]. In this study, the CCl_4_-induced mouse model was used to study the intervention effect of CBD on liver fibrosis and to explore whether its potential mechanism is related to the inhibition of NF-κB and activation of the PPAR-α signaling pathway and anti-inflammatory and antioxidant stress damage.

Increasingly, we found that CCl_4_-induced liver fibrosis resulted in significant weight loss, increased liver weight, and increased serum AST and HA levels. Pathological analysis of liver tissue in the CCl_4_ group showed massive liver cell necrosis, diffuse inflammatory cells and high levels of collagen, indicating that CCl_4_ caused severe liver damage. This study showed that CBD reduced the levels of AST and HA and reduced inflammatory infiltration and collagen deposition in liver tissues, indicating that CBD significantly alleviates CCl_4_-induced liver fibrosis, but there were similar effects between 4 mg/kg and 8 mg/kg CBD.

TGF-β1 is an essential cytokine that regulates the production, degradation and accumulation of ECM and plays a significant role in the activation of HSCs. In normal liver tissue, the expression of TGF-β1 in the liver is reduced. However, when liver injury occurs, HSCs, Kupffer cells (KCs) and other related cells produce large amounts of TGF-β1, which activates HSCs to form myofibroblasts, promotes the production of ECM and inhibits matrix degradation, resulting in the accumulation of scar matrix and liver fibrosis [50, 51]. Activated HSCs secrete large amounts of fibrillar collagens, mainly in the form of α-SMA and COL-I [52, 53], and the expression of the COL-I gene could indicate the synthesis of collagen. CBD inhibited the gene and protein expression of COL-I, α-SMA and TGF-β1. However, within the CBD concentration range set by the experiment, there was no statistically significant difference between the 4 mg/kg and 8 mg/kg CBD groups in various detection results. These results indicated that CBD inhibited collagen formation to prevent liver fibrosis, and further research is needed to determine the signaling pathways that mediate this protective effect on the liver.

Inflammation is closely related to the development of CCl_4_-induced liver fibrosis. IL-1β is one of the main factors inducing fibrosis, which promotes the aggregation of fibroblasts and inflammatory cells, as well as collagen and fibrin synthesis, which further leads to ECM deposition. We examined a series of inflammatory genes, including IL-6, IL-1β, and TNF-α, and found that CCl_4_ enhanced the expression of IL-6, IL-1β, and TNF-α. CBD blocked the inflammatory response in mouse liver tissue, which supported the anti-inflammatory effect of CBD, and the underlying mechanism may be associated with the inhibition of inflammatory signaling pathways.

NF-κB is an important nuclear transcription factor in the cell that participates in inflammatory and immune responses and can regulate cell apoptosis and the stress response [54, 55]. The phosphorylation of NF-κB inhibitory protein (IκB) enhanced the activity of NF-κB, promoted the nuclear transport of the NF-κB subunit, and triggered the transcription of downstream inflammatory genes, such as TNF-α, IL-6, and IL-1β. According to reports, activation of the NF-κB pathway can enhance the inflammatory response and EMT in liver cells. In the present study, we observed increased protein expression levels of total p-NF-κB, NF-κB, p-IκBα/IκBα, and downstream TNF-α, IL-6, and IL-1β in CCl_4_-induced mice. CBD treatment inhibited the activation of NF-κB signaling and the increases in these inflammatory cytokines, suggesting that CBD may inhibit NF-κB signaling and reduce the inflammatory response [56]. p38 MAPK mitogen-activated protein kinase belongs to the family of mitogen-activated protein kinases (MAPKs), which regulate the cell cycle, inflammation, growth, apoptosis, differentiation and other physiological processes. p38 MAPK can be phosphorylated by many extracellular agonists through the canonical MAPK pathway, and p-p38 MAPK can further regulate many substrates, such as transcription factors and PPARs [57]. PPAR belongs to the hormone nuclear receptor superfamily and consists of three subtypes (PPAR-α, PPAR-β/δ, and PPAR-γ) [58–60]. PPAR-α has important functions in regulating cells and is involved in cell proliferation, differentiation, oxidative/nitrification stress, inflammation and immune response. PPAR-α has been reported to reverse fibrosis by reducing lipid peroxidation and inhibiting activation of HSCs and KCs [61, 62]. Furthermore, studies have shown that inhibiting p-p38 MAPK increased PPAR-α expression to protect the liver against concanavalin A-induced injury [57]. We examined the expression of COX-2, p-p38/p38 and PPAR-α and demonstrated that CBD treatment reduced COX-2 expression, inhibited p-p38 MAPK, activated the PPAR-α signaling pathway, and protected the liver from fibrosis. These findings suggest that CBD can protect against liver fibrosis by activating the PPAR-α signaling pathway, and this study may make great progress in the treatment of chronic liver fibrosis.

In summary, we have shown that intraperitoneal injection of CBD exerts potent anti-inflammatory and antifibrotic activities *in vivo*. Moreover, we found that the first time CBD efficacy in reducing CCl_4_-induced hepatic fibrosis by multiple mechanisms. These mechanisms may involve inhibition of NF-κB, activation of the PPAR-α pathway, and inhibition of oxidative stress. Based on these findings, CBD has the potential to be further developed as a treatment for hepatic fibrosis, especially as a combination therapy with the currently available therapies.

## Data Availability

The original contributions presented in the study are included in the article/supplementary material, further inquiries can be directed to the corresponding author.
